# Occurence of RAS reversion in metastatic colorectal cancer patients treated with bevacizumab

**DOI:** 10.18632/oncotarget.27965

**Published:** 2021-05-25

**Authors:** Samantha Epistolio, Marco Cefalì, Paolo Spina, Francesca Molinari, Alessandra Movilia, Massimiliano Cergnul, Luca Mazzucchelli, Sara De Dosso, Milo Frattini, Piercarlo Saletti

**Affiliations:** ^1^Institute of Pathology, EOC, Locarno, Switzerland; ^2^Oncology Institute of Southern Switzerland, EOC, Bellinzona, Switzerland; ^3^Department of Health Sciences, University of Eastern Piedmont, Novara, Italy; ^4^Department of Pathology, ASST Ovest Milanese, Ospedale di Legnano, Legnano, Italy; ^5^Department of Medical Oncology, ASST Ovest Milanese, Ospedale di Legnano, Legnano, Italy; ^6^Current address: Department of Medical Oncology, Clinica Luganese Moncucco, Lugano, Switzerland; ^*^These authors are Joined First Authors; ^#^These authors are Joint Senior Authors

**Keywords:** RAS mutations, bevacizumab, metastatic colorectal cancer, next-generation sequencing

## Abstract

Background: A disappearance of RAS mutations in the plasma of about 50% of mCRCs (metastatic colorectal cancers) treated with bevacizumab-based chemotherapy has been reported. Our aim was to evaluate the same issue at tissue level.

Materials and Methods: Using next-generation sequencing and real-time PCR approaches, we characterized the primary tumor (PT) and paired liver metastases in 28 RAS mutant mCRCs. Patients were subdivided into 3 treatment groups: 1) bevacizumab plus chemotherapy; 2) chemotherapy alone; 3) any systemic therapy (control group). In groups 1 and 2, liver metastases were resected after removal of PT and subsequent neoadjuvant systemic therapy.

Results: RAS mutant alleles are at the same percentage in PT and liver metastases in the control group, while a significant reduction of the level of RAS mutations was detected in 57.1% of cases in group 1 and in 8.3% of cases in group 2. Differences among groups are statistically significant (*p* = 0.038).

Conclusions: Most of mCRC patients treated with bevacizumab-containing regimens experience a strong reduction of RAS mutant cells, suggesting bevacizumab as particularly active against RAS mutant cells. This finding might have potential therapeutic implications, as anti-EGFR could be reconsidered in primarily RAS mutant patients reverted to a wild-type status after bevacizumab exposure.

## INTRODUCTION

The overall survival (OS) for patients with metastatic colorectal cancer (mCRC) has markedly improved within the last 2 decades, reaching approximately 30 months [1–3].

The therapeutic options include fluoropyrimidines, oxaliplatin and irinotecan combinations (either doublets or triplets) with one anti-vascular endothelial growth factor (anti-VEGF) compound (i.e., bevacizumab, aflibercept and ramucirumab), and/or anti-epidermal growth factor receptor (EGFR) monoclonal antibody (MoAb, namely cetuximab or panitumumab) for patients with RAS/BRAF wild-type status, administered across different treatment lines [4]. Many factors contribute to the choice of the optimal treatment sequence, among these the tumor molecular status.

An important advancement in mCRC patients is based on a better understanding of both tumor biology and microenvironment. The RAS/RAF/MAPK pathway is downstream of EGFR, and its mutations are predictive for a lack of benefit from EGFR targeted therapies [5–9]. Thus, it is crucial to perform an extended RAS and BRAF mutation analysis before considering EGFR inhibitors in mCRC patients. In first-line, data support the use of anti-EGFR MoAbs in patients with left-sided, RAS and BRAF wild-type tumors, whilst bevacizumab is preferred in combination with chemo-doublets or -triplet in RAS/BRAF mutated tumors or RAS/BRAF wild-type right-sided primaries [10, 11].

More recently, two intriguing studies showed that cetuximab sensitivity might be restored either in RAS/BRAF wild-type mCRC patients who acquired resistance to cetuximab-based therapy in first-line therapy [12] or in those with primarily RAS mutated tumors treated with bevacizumab-containing regimens in second-line [13]. These studies may lead to the hypothesis that anti-angiogenic agents through action on RAS mutated cells could revert tumors from RAS mutant to RAS wild-type status, which theoretically could lead to the possibility to treat these patients with anti-EGFR MoAbs, otherwise precluded. In both mentioned trials, molecular analyses were performed by using liquid biopsies.

To substantiate these findings at tissue level, here we analyzed molecular changes in RAS mutated mCRC patients treated with chemotherapy alone or in combination with bevacizumab, by examining tumor tissue samples before and after the systemic therapy using two independent methodologies, next-generation sequencing (NGS) and real-time PCR.

## RESULTS

### Study population

The cohort included 28 patients (14 male, 14 female); median age at diagnosis was 67.6 years (range 39–87). All patients exhibited RAS mutation in primary tumor, located in 25 cases in the colon and in 3 cases in the rectum. The metastases were limited to the liver.

Our cohort is subdivided in three groups as previously described (details are summarized in Table 1).

**Table 1:** Details of the patients included in the three treatment groups analyzed by IOT

*n*	Group	Sex	Age	PT localization	LM0 (sy)	LM1 (me)	LM2 (me)	LM3 (me)
**1**	1	M	59	right colon		✓	✓	
**2**	1	M	70	left colon		✓	✓	
**3**	1	M	69	left colon		✓		
**4**	1	M	64	rectum		✓	✓	
**5**	1 + 3	F	61	right colon	✓	✓		
**6**	1	M	69	left colon		✓		
**7**	1	F	65	left colon		✓		
**8**	2	F	70	left colon		✓		
**9**	2 + 3	F	57	right colon	✓	✓		
**10**	2	F	58	right colon		✓		
**11**	2	F	71	left colon		✓		
**12**	2	M	48	right colon		✓		
**13**	2	F	75	right colon		✓		
**14**	2	M	76	rectum		✓	✓	
**15**	2	M	72	rectum		✓		
**16**	2	M	47	left colon		✓	✓	
**17**	2	F	81	right colon		✓		
**18**	2	F	58	right colon		✓	✓	✓
**19**	2	M	81	left colon		✓	✓	
**20**	3	F	39	right colon	✓			
**21**	3	M	61	left colon	✓			
**22**	3	M	78	left colon	✓			
**23**	3	F	68	left colon	✓			
**24**	3	M	83	right colon	✓			
**25**	3	F	85	right colon	✓			
**26**	3	F	75	right colon	✓			
**27**	3	F	87	left colon	✓			
**28**	3	M	66	right colon	✓			

Group 1: patients deemed potentially suitable for secondary liver resection after preoperative chemotherapy plus bevacizumab; Group 2: patients deemed potentially suitable for secondary liver resection after chemotherapy alone; Group 3: the control arm, i.e. patients with liver metastases deemed resectable without any systemic therapy. Abbreviations: M: male; F: female; LM0: synchronous liver metastasis; LM1: metachronous liver metastasis 1; LM2: metachronous liver metastasis 2; LM3: metachronous liver metastasis 3; me: metachronous; *n*: patient number; PT: primary tumour; sy: synchronous.

Group 1 consisted of 7 patients (5 male, 2 female) who received first-line chemotherapy plus bevacizumab before resection of liver metastases; the median age was 65.3 years (range 59–70).

Group 2 included 12 patients (5 male, 7 female) who underwent first-line chemotherapy alone before resection of liver metastases; the median age was 66.2 years (range 47–81). In Groups 1 and 2 liver metastases were resected metachronously with respect to the primary tumor, at completion of systemic therapy.

The Group 3, control arm, included 11 patients (4 male, 7 female) who did not receive systemic therapy because they underwent concomitant resection of the primary tumor and paired liver metastases. The median age in this group was 69.5 years (range 39–87).

Two cases, out of the 28 included in our cohort, were classified in two distinct groups, namely patient 5 in Groups 1 and 3 and patient 9 in Groups 2 and 3, as they underwent concomitant resection of liver metastases with primary tumor but also secondary resection after systemic therapy.

In some patients we had the opportunity to analyze metastases that emerged later during the follow-up after initial multimodality therapy. In 3 cases of Group 1 (patients 1, 2 and 4) two metastases were available. In Group 2, two metastases were available in 3 cases (patients 14, 16 and 19) and 3 metastases in one case (patient 18).

### Assessment of the uniformity of tissue samples: preliminary experiment

We initially selected two cases, patients 8 and 9 (Table 1), for whom abundant tumor tissue was available for molecular analyses. For each patient we examined by NGS and real time PCR 4 different formalin-fixed paraffin-embedded (FFPE) tissue block from the primary tumor (PT, namely PTa, PTb, PTc, PTd) and 4 different FFPE blocks from the liver metastasis (LM, namely LM1a, LM1b, LM1c, LM1d). All details are summarized in Table 2.

**Table 2:** Results of the pilot experiment to assess the uniformity of the tissue samples

*n*		Sample	Group	Localization	KRAS-(%)	APC-(%)	TP53-(%)	PIK3CA-(%)
**Patient 8**	PT Patient 8 (4 different FFPE blocks)	8PTa	2	left colon	G13D (45.7%)	T1556fs (63.6%)	wt	E545K (43%)
		8PTb	2	left colon	G13D (47.6%)	T1556fs (65.6%)	wt	E545K (41.8%)
		8PTc	2	left colon	G13D (55.6%)	T1556fs (63.8%)	wt	E545K (42.6%)
		8PTd	2	left colon	G13D (52.6%)	T1556fs (62.6%)	wt	E545K (40.3%)
	LM1 Patient 8 (4 different FFPE blocks)	8LM1a	2	liver	G13D (57.6%)	T1556fs (57.9%)	wt	E545K (42%)
		8LM1b	2	liver	G13D (48.7%)	T1556fs (60.5%)	wt	E545K (38.7%)
		8LM1c	2	liver	G13D (50.2%)	T1556fs (63%)	wt	E545K (43.2%)
		8LM1d	2	liver	G13D (41.8%)	T1556fs (61.6%)	wt	E545K (42.8%)
**Patient 9**	PT Patient 9 (4 different FFPE blocks)	9PTa	2	right colon	G12V (34.7%)	wt	wt	E542K (51.6%)
		9PTb	2	right colon	G12V (35.7%)	wt	wt	E542K (49.2%)
		9PTc	2	right colon	G12V (33.3%)	wt	wt	E542K (50.8%)
		9PTd	2	right colon	G12V (32%)	wt	wt	E542K (52.4%)
	LM1 Patient 9 (4 different FFPE blocks)	9LM1a	2	liver	G12V (36.6%)	wt	wt	E542K (50.1%)
		9LM1b	2	liver	G12V (32.4%)	wt	wt	E542K (54%)
		9LM1c	2	liver	G12V (34.2%)	wt	wt	E542K (51.8%)
		9LM1d	2	liver	G12V (36%)	wt	wt	E542K (49.5%)

Four different tissue blocks of patients 8 and 9 were selected for the molecular characterization. Number in brackets in the gene columns are the result of the percentages obtained from IOT experiments normalized on tumor cell content. Abbreviations: FFPE: Formalin-Fixed Paraffin-Embedded; fs: frame-shift; IOT: Ion Torrent; LM1: metachronous liver metastasis 1; *n:* patient number; PT: Primary Tumour; wt: wild-type.

After normalization based on the tumor content evaluated by the pathologists, the mutations detected in different blocks led us to establish that patient 8 exhibits the same mutations at similar percentages in all areas of the PT (samples 8PTa, 8PTb, 8PTc, 8PTd) and in all different regions of paired liver metastasis (samples 8LM1a, 8LM1b, 8LM1c, 8LM1d). Similar findings were observed in patient 9 after analysis of PT (samples 9PTa, 9PTb, 9PTc, 9PTd) and of the metachronous metastasis (samples 9LM1a, 9LM1b, 9LM1c, 9LM1d). The comparison data concerning the analysis of different tumor areas were not obtained for the synchronous metastasis of patient 9 (i.e.,: 9LM0) because only one tissue block was available (Table 3).

**Table 3:** Ion Torrent patterns in primary tumour and in the different liver metastases

*n*	Treatment group	Sample number	RAS-(%)	APC-(%)	TP53-(%)	Other genes-(%)
**1**	1	1PT	KRAS G12C (15.2)	wt	R280T (50.7)	PIK3CA: E542K (23.0)
		1LM1	KRAS G12C (6.8)	wt	R280T (20.0)	PIK3CA: E542K (12.6)
		1LM2	KRAS G12C (8.1)	wt	R280T (36.4)	PIK3CA: E542K (6.44)
**2**	1	2PT	KRAS G12V (24.8)	wt	wt	wt
		2LM1	KRAS G12V (8.1)	wt	wt	wt
		2LM2	KRAS G12V (32.9)	wt	wt	wt
**3**	1	3PT	KRAS G12S (23.8)	R1114^*^ (34.4)	R282W (35.1)	wt
		3LM	KRAS G12S (37.9)	R1114^*^ (59.9)	R282W (64.4)	PIK3CA: E542K (23.0)
**4**	1	4PT	KRAS G12V (25.9)	E1379^*^ (46.9)	F134L (45.9)	SMAD4: D355V (25.7)
		4LM1	KRAS G12V (7.9)	E1379^*^ (15.2)	F134L (11.8)	SMAD4: D355V (7.1)
		4LM2	KRAS G12V (12.7)	E1379^*^ (21.2)	F134L (13.9)	SMAD4: D355V (16.8)
**5**	1 + 3	5PT	KRAS G12C (38.3)	wt	R273H (50.8)	wt
	3	5LM0	KRAS G12C (34.1)	wt	R273H (52.2)	wt
	1	5LM1	KRAS G12C (32.6)	wt	R273H (35.1)	wt
**6**	1	6PT	NRAS G12D (21.2)	Q1291^*^ (61.0)	T256P (37.8)	PIK3CA: E542K (10.2)
	1	6LM1	NRAS G12D (6.9)	Q1291^*^ (15.5)	T256P (8.8)	wt
**7**	1	7PT	KRAS G12D (22.2)	E1306K (19.3)	wt	wt
		7LM1	KRAS G12D (14.41)	E1306K (8.8)	wt	wt
**8**	2	8PT	KRAS G13D (45.7)	T1556fs (63.6)	wt	PIK3CA E545K (43.0)
		8LM1	KRAS G13D (57.6)	T1556fs (57.9)	wt	PIK3CA E545K (42.0)
**9**	2 + 3	9PT	KRAS G12V (34.7)	wt	wt	PIK3CA E542K (51.6)
	3	9LM0	KRAS G12V (35.3)	wt	wt	wt
	2	9LM1	KRAS G12V (36.6)	wt	wt	PIK3CA E542K (50.1)
**10**	2	10PT	KRAS G12S (40.1)	wt	wt	CDKN2A: V106M (39.6)
		10LM1	KRAS G12S (38.6)	wt	wt	CDKN2A: V106M (25.5)
**11**	2	11PT	KRAS G13D (16.8)	G1499^*^ (15.9)	wt	wt
		11LM1	KRAS G13D (8.4)	G1499^*^ (12.5)	wt	wt
**12**	2	12PT	KRAS G12S (43.7)	S1503^*^ (24.8)	wt	PIK3CA: C420R (18.2)
		12LM1	KRAS G12S (41.1)	S1503^*^ (22.9)	wt	PIK3CA: C420R (20.4)
**13**	2	13PT	KRAS G12V (16.2)	I1307Kfs^*^8 (42.6)	C242fs^*^5 (29.8)	wt
		13LM1	KRAS G12V (20.9)	I1307Kfs^*^8 (52.4)	C242fs^*^5 (28.7)	wt
**14**	2	14PT	KRAS G13D (27.2)	Q1378^*^ (28)	Y220C (42.1)	wt
		14LM1	KRAS G13D (23.9)	Q1378^*^ (18.4)	Y220C (27.0)	SMAD4: R135^*^ (14.5)
		14LM2	KRAS G13D (19.9)	Q1378^*^ (19.1)	Y220C (28.1)	wt
**15**	2	15PT	KRAS G12D (13.3)	wt	wt	wt
		15LM1	KRAS G12D (13.1)	wt	wt	wt
**16**	2	16PT	KRAS G12D (21.6)	G1357^*^ (23.8)	R175H (23.0)	wt
		16LM1	KRAS G12D (16.8)	G1357^*^ (30.8)	R175H (35.4)	wt
		16LM2	KRAS G12D (29.0)	G1357^*^ (39.7)	wt	wt
**17**	2	17PT	KRAS G12D (12.4)	wt	wt	CDKN2A: D84N (16.2)
		17LM1	KRAS G12D (18.8)	wt	wt	CDKN2A: D84N (14.5)
**18**	2	18PT	KRAS G12A (21.9)	wt	H214R (33.8)	wt
		18LM1	KRAS G12A (22.1)	wt	H214R (47.2)	wt
		18LM2	KRAS G12A (17.6)	wt	H214R (9.4)	wt
		18LM3	KRAS G12A (33.2)	wt	H214R (21.7)	wt
**19**	2	19PT	KRAS G12A (46.4)	wt	P151T (61.6)	wt
		19LM1	KRAS G12A (41.5)	wt	P151T (76.2)	wt
		19LM2	KRAS G12A (48.7)	wt	P151T (63.5)	wt
**20**	3	20PT	KRAS G13D (32.5)	wt	P98S (33.8)	EGFR: V774M (34.2)
		20LM0	KRAS G13D (24.5)	wt	P98S (29.1)	EGFR: V774M (23.2)
**21**	3	21PT	KRAS G12D (54.7)	F1491Lfs^*^16 (38.9)	wt	wt
		21LM0	KRAS G12D (44.8)	F1491Lfs^*^16 (37.1)	wt	wt
**22**	3	22PT	KRAS G12C (22.9)	wt	wt	wt
		22LM0	KRAS G12C (18.1)	wt	wt	wt
**23**	3	23PT	KRAS G12D (29.4)	E1309Dfs^*^4 (45.3)	wt	SMAD4: W398^*^ (49.7)
		23LM0	KRAS G12D (20.6)	E1309Dfs^*^4 (42.2)	wt	SMAD4: W398^*^ (35.6)
**24**	3	24PT	KRAS G12D (49.6)	wt	E271^*^ (54.4)	PTEN: Y240^*^ (63.1); GNAS: R844C (3.0)
		24LM0	KRAS G12D (56.2)	wt	E271^*^ (61.2)	PTEN: Y240^*^ (56.2); GNAS: wt
**25**	3	25PT	KRAS G12C (51.3)	wt	R196^*^ (37.0)	PIK3CA: E453K (40.4)
		25LM0	KRAS G12C (50.5)	wt	R196^*^ (69.8)	PIK3CA: E453K (32.4)
**26**	3	26PT	KRAS G12D (28.4)	wt	wt	RB1: D340N (6.9)
		26LM0	KRAS G12D (30.9)	wt	wt	RB1: D340N (6.5)
**27**	3	27PT	KRAS G12D (41.2)	P1439Lfs^*^34 (31.6)	Q136^*^ (64.6)	wt
		27LM0	KRAS G12D (39.7)	P1439Lfs^*^34 (46.7)	Q136^*^ (65.8)	wt
**28**	3	28PT	NRAS Q61K (32.2)	wt	wt	PIK3CA: E453K (40.4)
		28LM0	NRAS Q61K (35.9)	wt	wt	PIK3CA: Q546K (28.0)

In brackets IOT percentages of the mutations normalized on the percentage of tumour cell content are reported. Abbreviations: IOT: Ion Torrent; LM0: synchronous liver metastasis; LM1: metachronous liver metastasis 1; LM2: metachronous liver metastasis 2; LM3: metachronous liver metastasis 3; *n*: patient number; PT: Primary Tumour; wt: wild-type.

Overall, the findings obtained from patients 8 and 9 suggested that the mutations are uniformly distributed in both PT and paired liver metastases. Based on this we speculated that only one tissue block from the PT and from the paired liver metastases could reflect the molecular patterns of the whole tumor.

### IOT analysis of RAS and other relevant genes in primary tumor and paired liver metastases

In all cases, NGS experiments by Ion Torrent (IOT) provided the calculation of the percentage of the mutant allele of 48 genes in addition to KRAS and NRAS. The data obtained after analysis of PT and liver metastases tissue blocks were normalized with the tumor cells (*t* cells) % content.

### Group 1: patients treated with chemotherapy plus bevacizumab before resection of liver metastases

The comparison of RAS mutation percentages between PT and paired liver metastases resected after systemic therapy showed a reduction of at least 50% of RAS mutant alleles in 4 out of 7 cases (57.1%; patients 1, 2, 4 and 6); in 2 cases (patients 4 and 6) the decrease was greater than 3 times. In the remaining cases, a heterogeneous trend was observed, with a decrease of about 30% in 1 case (patient 7), similar rate in one case (patient 5) and an increase of 30% in patient 3 (Table 3).

In 4 cases, two liver metastases were available. In patient 5, a liver metastasis was synchronous to the PT and showed the same RAS mutant allele content as compared to PT. In patients 1 and 4, the two lesions resected after systemic therapy showed similar trends, with a reduction greater than 50% of RAS mutant allele content in both cases, as compared to the RAS mutant allele content in PT. In patient 2, the two liver metastases were not resected at the same time. The first one, removed immediately after systemic therapy, showed a more than 50% reduction of the RAS mutant allele content. The second metastasis, resected 8 months after initial therapy, showed an increase of 400% of RAS mutant alleles compared to the first liver metastasis, and even an increase of about 30% as compared to the PT.

Concerning the other mutations included in the NGS panel, we observed the same trends of RAS alterations with exception of patient 3 (in whom a new alteration in PIK3CA was detected in the metastasis) and patient 6 (the PIK3CA mutation was not seen in the metastasis).

### Group 2: patients treated with chemotherapy alone before resection of liver metastases

The comparison of RAS mutation frequencies between PT and liver metastases showed a reduction of at least 50% of RAS mutant allele content in only one patient (8.3%, patient 11, Table 3). All other cases showed RAS mutant allele frequencies nearly comparable between the PT and liver metastasis. Two or more liver metastases were available in 5 patients: in patient 9, one liver metastasis was synchronous to the PT; in patients 14, 16, 18 and 19 the metastases were resected with an interval of about 6–8 months and after second or third-line therapies not including bevacizumab. In all the cases, the RAS mutant allele content in metastases was similar to the PT, with a trend to increase in late resected metastases (Table 3).

As regards the other mutations included in the IOT panel, we observed the same trend of RAS alterations with few exceptions. Patient 11 did not experience a reduction of APC mutation percentage; patient 14 showed both APC and TP53 reduction and occurrence of SMAD4 frame-shift mutation in the first metastasis only; patient 16 had a higher percentage of APC mutant content and became wild-type in TP53 gene in the second metastasis; patient 18 presented a trend of decrease in TP53 mutation rates starting from the second metachronous metastasis (Table 3).

### Group 3 (control group): patients with resected liver metastasis without any systemic therapy

As expected, all the samples of the control group (11/11; 100%) showed the same RAS mutant allele content in PT and synchronous liver metastasis (Table 3).

Concerning the other mutations included in the Cancer Hotspot Panel v2 (CHPv2), we confirmed the same trend of RAS alterations with few exceptions. Patient 9, who did not show PIK3CA mutations in the synchronous metastasis compared to the PT; patient 24, in whom GNAS mutation was not detected in the metastasis; patient 25, who showed a TP53 mutation increase; patient 28 who showed a decrease of PIK3CA mutant allele content in the metastasis (Table 3).

### Real-time analysis of RAS

**Figure 1:**
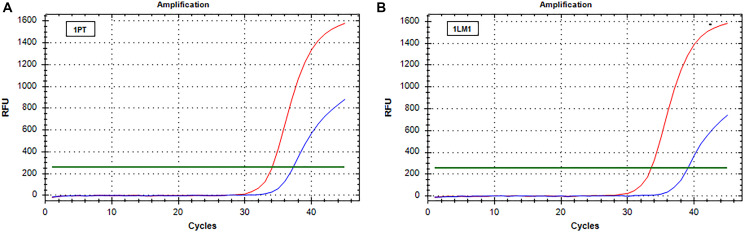
Real-time PCR (SensiScreen™) amplification curves of patient 1 (group 1). X-axis reports real-time PCR cycles and Y-axis reports relative fluorescence unit (RFU). In red is represented the amplification of the reference gene and in blue the amplification of the specific mutation (G12C). (**A**) Curves obtained from amplification of DNA extracted from sample 1PT; (**B**) Curves obtained from amplification of DNA extracted from sample 1LM1. Abbreviations: 1LM1, liver metastasis sample (patient 1); 1PT, primary tumor sample (patient 1); RFU, relative fluorescence unit.

**Figure 2:**
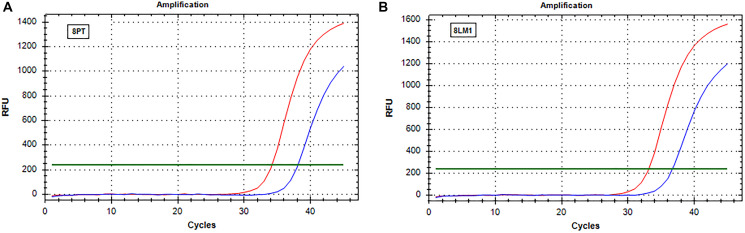
Real-time PCR (SensiScreen™) amplification curves of patient 8 (group 2). X-axis reports real-time PCR cycles and Y-axis reports relative fluorescence unit (RFU). In red is represented the amplification of the reference gene and in blue the amplification of the specific mutation (G13D). (**A**) Curves obtained from amplification of DNA extracted from sample 8PT; (**B**) Curves obtained from amplification of DNA extracted from sample 8LM1. Abbreviations: 8LM1, liver metastasis sample (patient 8); 8PT, primary tumor sample (patient 8); RFU, relative fluorescence unit.

The real-time data obtained after analysis of PT and liver metastases tissue blocks were normalized with the *t* cells % content. Real-time results mirror those obtained by IOT. In particular, when a decrease of RAS mutations level was observed by IOT, real-time showed an increase of ΔCt comparing PT and the associated metastases. On the contrary, the ΔCt by real-time was similar in patients showing no difference in RAS mutation frequency. An example of increased ΔCt is reported in Figure 1 and an example of stability of ΔCt in Figure 2.

### Statistical analyses

The evaluation of RAS mutant allele content variations in Groups 1 and 2 showed a significant correlation (*p* ≤ 0.05) between bevacizumab administration and the decrease of more than 50% of RAS mutations by applying the two-tailed Fisher’s exact test (Table 4).

**Table 4:** Two-tailed Fisher exact test results

	RAS % (comparison between PT and LM)	
	Comparable	Decrease of at least 50%
Chemotherapy plus bevacizumab (group 1)	3	4
Chemotherapy (group 2)	11	1
		*p* = 0.038

Level of significance: *p* < 0.05. Abbreviations: LM: Liver Metastases; PT: Primary Tumour.

## DISCUSSION

In patients with mCRC, two preliminary studies reported that bevacizumab could revert RAS mutant status, assessed by liquid biopsies [12, 13].

In the CRICKET trial, twenty-seven RAS wild type mCRC patients were successfully treated for at least 6 months with cetuximab plus chemotherapy. At disease progression, patients underwent second line bevacizumab-containing regimens. In those who experienced a benefit from the second line for at least 4 months, an anti-EGFR rechallenge was proposed at progression. Four cases (14.3%) showed a partial response, with additional 15 cases (53.5%) achieving a disease control. The median progression-free survival (PFS) and median OS were 3.4 months (95% CI: 1.9–3.8 months) and 9.8 months (95% CI: 5.2–13.10 months), respectively [14]. The investigators evaluated RAS status in liquid biopsies collected at progression during bevacizumab and before the EGFR rechallenge, and found that all patients who exhibited a confirmed partial response were RAS wild type.

In another study, eleven RAS mutant mCRC patients were treated with first line bevacizumab-based regimens. Interestingly, the authors reported that at disease progression the RAS status at liquid biopsies reverted to a wild-type pattern in 6 patients (55.5%), and patients were successfully treated with cetuximab (PFS ranging from 6 to 12 months in those treated in second-line, PFS of 4 months in a patient treated in fourth line) [13, 15]. Taken together, these data suggest that bevacizumab is more effective against RAS mutant cells, possibly even leading to a conversion from RAS mutant to RAS wild-type status, at least when evaluated by liquid biopsy.

To date, liquid biopsy represents a promising source of genomic DNA that can be used for diagnostic purposes. The application of liquid biopsies is accepted in the management of patients with non-small cell lung cancer (NSCLC), but its role in CRC is still marginal. If on the one hand liquid biopsy may provide a dynamic characterization of the mutational status over time, the analysis of samples requires techniques characterized by a very low limit of detection (LOD), equal or less than 0.1% because the mutant alleles are extremely diluted in blood vessels, thus could be potentially classified as false wild type. The concordance between tumor tissue and liquid biopsy is only observed in highly vascularized tumors. For example, in NSCLC the concordance is up to 70%, while in CRC concordance reaches values above 90% [16–18].

In our study we aimed to assess whether exposure to bevacizumab could revert a RAS mutant tumor to RAS wild-type status, or at least induce a significant decrease of RAS mutant *t* cells at tissue level in patients with mCRC. To explore this concept, we identified three cohorts of previously untreated patients with RAS mutated mCRC, treated either with (Group 1) or without (Group 2) bevacizumab-based regimens before resection of liver metastases. The control arm (Group 3) consisted of patients with RAS mutant tumors, who underwent resection of liver metastases without any systemic preoperative therapy. Before the analyses of the whole cohort, in two cases selected on the basis of availability of multiple tissue blocks of both PT and paired liver metastases, we evaluated the RAS mutant allele content in different portions of the two lesions. After normalization with the t cell content provided by the pathologists, we found that RAS mutations were distributed homogeneously in the PT as well as in the liver metastasis. Thus, we can assume that the analysis of a single tissue block is representative of the totality of the malignancy and we can be confident in analysing only one block in the patients of the aforementioned three groups.

In the control arm (group 3) we observed 100% concordance of RAS mutant allele content between PT and liver metastases. This finding suggests that there is no clonal selection for RAS mutant cells in the natural history of mCRC patients on the one hand, and that cells with RAS mutations are not more aggressive than RAS wild-type cells on the other hand, confirming the absence of correlation between RAS mutation and worse prognosis [19]. Then we focused our attention to patients treated with chemotherapy alone before resection of liver metastases. Here we observed a similar pattern with respect to the control group. Indeed, only 1 out of 12 cases shows a drop of more than 50% of RAS mutant cell content, suggesting that chemotherapy alone is effective independently from the RAS mutational status. The situation is completely different in patients treated with a bevacizumab-containing regimen. In the majority of cases (4 out of 7 patients) we observed a dramatic decrease of RAS mutant allele content (at least 50%) in liver metastasis with respect to the PT resected before systemic therapy. Interestingly, results changed in the patient who underwent resection of additional liver metastases after a second line systemic therapy not including bevacizumab. This single case presented an increase of 400% of the RAS mutant allele content compared to RAS mutant allele observed in liver metastases removed at the end of a bevacizumab-containing regimen, even greater than the value observed in the PT. The difference in the decrease of the RAS mutant allele content between PT and liver metastasis after systemic therapy was statistically significant when Group 1 and Group 2 are compared (*p* = 0.038). In line with previously mentioned studies using liquid biopsies [13–15], our results overall confirm that bevacizumab-based systemic therapy seems to reduce RAS mutant cells burden, even at the tissue level. In contrast with these studies, in our cohort we did not observe a complete regression of RAS mutant cells in liver metastases. The differences may be explained by methodological issues: indeed the assays used for molecular characterization of plasma samples, including also the IOT panels for plasma analyses, are usually not sensitive enough to identify very low copy numbers of the mutant allele. It is therefore not surprising that our results show a significant decrease but not complete disappearance of RAS mutant cells, especially taking into account that bevacizumab is not a RAS-targeted agent. How bevacizumab could exerts its effect on RAS status changes still needs to be elucidated, but inflammation and neo-angiogenesis can be taken in consideration, as suggested in studies on transgenic murine models [20–22].

The precise estimation of the RAS mutant allele content in tumor tissues is challenging. In particular, the t cell content represents a crucial factor for such analysis because the RAS mutant allele content is provided by pathologists, based on a subjective histological estimation. The comparison of tissue samples from the same patient and the evaluation performed by different skilled pathologists should minimize inter-observer variability and interpretation bias. In our study two pathologists with experience in gastrointestinal tract cancer examined cases and provided an estimated t cell content in a blinded manner. As the concordance between pathologists was 100%, the subsequent use of a semi-quantitative method for the molecular characterization (IOT CHPv2 panel) should provide a percentage of the mutant allele truly reflecting the real content. To substantiate our evaluation, we also applied another semi-quantitative method (real-time PCR-based) for the analysis of RAS mutations, confirming the results obtained by the NGS analysis. The overlapping of NGS results and real-time PCR data observed in our work has been also described in literature. Indeed, a relevant number of papers, concerning KRAS evaluation in CRC and NSCLC samples, reports a significant concordance (from 93 to 100%) between these two methodologies [23–25].

The main limitation of our study is represented by the limited number of patients. Thus, our findings deserve to be confirmed in larger and independent cohorts.

In conclusion, this retrospective observational study, strongly suggests the existence of a link between bevacizumab exposure and RAS status changes in mCRC patients. In addition to other reports using liquid biopsies, our findings on tissue samples corroborate the hypothesis that bevacizumab could revert RAS mutant mCRC to a wild-type pattern, conceptually opening to the possibility to treat with anti-EGFR MoAbs mCRC patients otherwise excluded based on initial RAS mutated status.

## MATERIALS AND METHODS

### Patients

All patients included in this retrospective study were treated in two institutions, the *Oncology Institute of Southern Switzerland* (Switzerland) and the Hospital of Legnano (Italy), from 2011 to 2019. Inclusion criteria were: age ≥ 18 years, histology proven mCRC, presence of mutations in RAS genes in primary tumor or paired resected liver metastases, availability of tumor tissue before and after systemic therapy. Exclusion criteria were: insufficient amount of *t* cells (< 10%), inadequate material for molecular purposes on primary tumor and paired liver metastasis.

Our study population cohort consisted of three groups: 1) patients deemed potentially suitable for secondary liver resection after preoperative chemotherapy plus bevacizumab; 2) patients deemed potentially suitable for secondary liver resection after chemotherapy alone; 3) the control group, namely patients with liver metastases deemed resectable without any systemic therapy.

As most of these patients were deceased before entry in this retrospective study, the permission to perform the molecular analyses was given by the Regional Ethical Committee (No.: 2019-02224). For those who were alive at study entry a signed informed consent was requested. The study was conducted in accordance with the Declaration of Helsinki.

### Molecular analyses

All tumor tissue specimens were evaluated by two senior pathologists of the Institute of Pathology in Locarno (Switzerland). The pathologists selected the appropriate tumor area on hematoxylin and eosin (HE) stained slides, and independently established the *t* cells %. Genomic DNA was extracted from six 7 μm-thick serial sections of each selected FFPE tissue block from the primary tumor and paired liver metastasis using the QIAamp DNA FFPE Tissue kit (Qiagen, USA), according to the manufacturer’s instructions. DNA was analyzed using an NGS approach on S5 IOT platform, by applying the Ion AmpliSeq™ Cancer Hotspot Panel v2 (CHPv2) (ThermoFisher Scientific, USA) [26, 27]. The CHPv2 panel generated semi-quantitative and qualitative data on the mutational status of RAS and 49 additional genes, encompassing the most relevant and frequently mutated genes in CRC (i.e., APC, TP53, PIK3CA, BRAF and PTEN). NGS data give an idea of how many cells of the sample are mutated in each gene, through the evaluation of the Variant Allele Frequency (VAF) of each mutation. The quantitative level of RAS mutations in both primary tissue and liver metastasis was obtained by normalizing VAF of RAS mutation obtained from the NGS experiment with the *t* cells % calculated by the pathologists. The presence of RAS mutations was also confirmed quantitatively using an independent methodology based on a real-time PCR amplification (SensiScreen, PentaBase ApS, Denmark) [28]. Real-time data were evaluated comparing ΔCt values of PT and paired liver metastasis. ΔCt was calculated as the difference between the threshold cycle (Ct) of reference gene and the Ct of the specific RAS mutation previously detected by IOT.

### Statistical analyses

The loss of RAS mutations was considered meaningful in presence of a difference greater than 50% between the primary tumor and paired liver metastases. The two-tailed Fisher’s exact test was used to calculate *p*-values and the level of significance was set at *p* < 0.05.

### Ethics approval and consent to participate

This work has been conducted in compliance with the current version of the Declaration of Helsinki as well as all national legal and regulatory requirements. Data and samples have been collected and analyzed for the study purpose only after the required authorizations from the competent Ethics Committees were obtained (No.: 2019-02224). As most patients has deceased before study entry, the permission to perform the molecular analyses was given by the Regional Ethics Committee. Conversely, for those who were alive at study entry a signed informed consent was requested. To partecipate in this study a consent for the publication of the anonymized data has also been obtained.

All the data reported in this paper have been anonymized and they have been obtained following the national legal and regulatory requirements.

## Abbreviations

EGFR: epidermal growth factor receptor
FFPE: formalin fixed paraffin embedded
HE: hematoxylin and eosin staining
MoAbs: monoclonal antibodies
mCRC: metastatic colorectal cancer
NGS: next-generation sequencing
NSCLC: non-small cell lung cancer
ORR: overall response rate
OS: overall survival
PFS: progression-free survival
PT: primary tumor
*t* cells: tumor cells
VEGF: vascular endothelial growth factor
